# Pre- and Post-bronchodilator Spirometry in Asthmatic Smokers Versus Non-smokers: A Hospital-Based Cross-Sectional Study

**DOI:** 10.7759/cureus.93986

**Published:** 2025-10-06

**Authors:** Yassine Wael Mohamed Elshammaa, Dana Mhd Nabil Bendakji, Sherif Elseedy, Mohammed Faeq, Mostafa Afifi, Ahmed Yehia Zakaria Khaled, Ali Abdallah, Basma Alleelwa, Toka Ghazy, Mohamed Nasr, Mohamed Ashraf Hussien Abdelhady, Naglaa Abdelsamea, Ahmed Mamdouh Elshafei Khaled, Afnan Elwafi, Mohsen Mosallam, Ibtihal Khider Fagir Salih, Nermeen Hassan, Marwa Harb, Mohamed Abdelfattah, Ahmed Mostafa, Abdulmabod Omar

**Affiliations:** 1 Faculty of Medicine, Alexandria University, Alexandria, EGY; 2 Acute Medicine, Aneurin Bevan University Health Board, Newport, GBR; 3 Medicine, University Hospital Hairmyres, NHS Lanarkshire, Glasgow, GBR; 4 Critical Care, King’s College Hospitals NHS Foundation Trust, London, GBR; 5 Faculty of Medicine, Al-Azhar University, Cairo, EGY; 6 Faculty of Medicine, Assiut University, Assiut, EGY; 7 Acute Medicine, Stockport NHS Foundation Trust, Stockport, GBR; 8 General Practice, Princess of Wales Hospital, Bridgend, GBR; 9 Faculty of Medicine, Mansoura University, Mansoura, EGY; 10 Faculty of Medicine, Ain Shams University, Cairo, EGY; 11 Respiratory Medicine, Blackpool Victoria Hospital, Blackpool Teaching Hospitals, NHS Foundation Trust, Blackpool, GBR; 12 Paediatrics, Ormskirk Mersey West Lancashire, NHS Foundation Trust, Ormskirk, GBR; 13 Acute Medicine, Grange University Hospital, Cwmbran, GBR; 14 Faculty of Medicine, Cairo University, Cairo, EGY; 15 General Practice, University Hospitals of Derby and Burton NHS Foundation Trust, Derby, GBR; 16 Endocrinology, Scarborough General Hospital, Scarborough, GBR; 17 Accident and Emergency, West Middlesex University Hospital Isleworth, London, GBR; 18 Hassan Ghazzawi Hospital, Abeer Medical Group, Jeddah, SAU

**Keywords:** airway obstruction, asthma, bronchodilator response, fev1/fvc ratio, lung function, smoking, spirometry

## Abstract

Background

Cigarette smoking is a major risk factor for impaired lung function and the development of chronic respiratory diseases. Early detection of pulmonary changes using spirometry can help in timely intervention and prevention of long-term complications.

Objective

To compare spirometry parameters between smokers and non-smokers, and to evaluate the effect of bronchodilator administration on pulmonary function in both groups.

Methods

A cross-sectional study was conducted involving 60 participants (30 smokers and 30 non-smokers). Spirometry was performed before and after bronchodilator administration to measure vital capacity (VC%), forced vital capacity (FVC%), forced expiratory volume in the first second (FEV₁%), FEV₁/FVC ratio, forced expiratory flow over the middle one half of the FVC (FEF25-75%), maximal voluntary ventilation (MVV%), and expiratory time. Paired t-tests were used for within-group comparisons, and independent t-tests for between-group comparisons. A p-value < 0.05 was considered statistically significant.

Results

Smokers had significantly lower baseline spirometry values compared to non-smokers across all parameters (p < 0.001). Both groups showed statistically significant improvements post-bronchodilator (p < 0.001), with smokers exhibiting a smaller magnitude of improvement. Notably, FEV₁% increased from 47.2 ± 6.8 to 60.3 ± 11.5 in smokers and from 56.3 ± 7.1 to 73.3 ± 7.4 in non-smokers. The FEV₁/FVC ratio and other flow rates showed similar patterns. Expiratory time also increased post-bronchodilator in both groups.

Conclusion

Smoking is associated with marked reductions in lung function and diminished bronchodilator responsiveness. Early spirometric screening in smokers is essential to identify functional decline and guide smoking cessation and treatment strategies to prevent progression of pulmonary disease.

## Introduction

One of the main causes of respiratory morbidity and mortality globally is still prolonged exposure to tobacco smoke, which also plays a major role in the development of other pulmonary illnesses, including chronic obstructive pulmonary disease (COPD) [1,2]. Smoking causes intricate pathophysiological alterations in the lung parenchyma and airways, which lead to tissue remodelling, inflammation, and restricted airflow, all of which gradually deteriorate lung function [3]. For identifying and tracking obstructive airway illnesses, spirometry (a non-invasive and widely available pulmonary function test) remains the gold standard. It examines a number of parameters that indicate the degree of airflow blockage and lung capacity, including forced vital capacity (FVC), forced expiratory volume in one second (FEV₁), and FEV₁/FVC ratio [4].

Despite the well-established negative effects of smoking on lung function, new research shows that even asymptomatic smokers with intact spirometric readings may develop early-stage lung damage [5]. These people may have subclinical airway abnormalities and a rapid deterioration in lung function, which are frequently missed by conventional diagnostic standards that mainly use spirometric thresholds to diagnose COPD [6]. This emphasises the urgent need for a thorough evaluation that includes bronchodilator responsiveness as well as other spirometric indicators like maximum voluntary ventilation (MVV) and forced expiratory flow over the middle one half of the FVC (FEF25-75), which may identify minute alterations in small airway function and respiratory muscle performance. Furthermore, designing successful therapeutic therapies requires an understanding of how smoking differs in its effects on lung function metrics before and after bronchodilators [7]. Since bronchodilator response can reveal information about inflammation, airway hyperresponsiveness, and reversible airway obstruction, all of which have consequences for therapy and prognosis [8]. Nevertheless, there is still a lack of information comparing these metrics between smokers and non-smokers in a variety of demographics, particularly in environments where smoking prevalence is still high and access to healthcare may be constrained.

The purpose of this study is to close these gaps by thoroughly assessing spirometric parameters in smokers and non-smokers before and after bronchodilators, either long-acting, short-acting beta agonists or anticholinergics. Vital capacity (VC), FVC, FEV₁, FEV₁/FVC ratio, FEF25-75, MVV, and expiratory time are all measures that we use to determine the degree and type of lung function impairment caused by smoking. This method will offer a thorough description of the alterations in pulmonary function brought on by smoking, possibly revealing early indicators of respiratory dysfunction. In the final analysis, the results of this study should help physicians optimise their diagnostic and treatment methods by providing insightful information on the early identification and treatment of smoking-related lung disorders. Furthermore, this study highlights the critical need for improved smoking cessation initiatives and healthcare prevention strategies to lessen the long-term respiratory health risks associated with tobacco use.

## Materials and methods

Study design and setting

To evaluate pulmonary function in adult asthmatic patients according to cigarette smoking status, a cross-sectional study was carried out at Hassan Ghazzawi Hospital. There were 60 volunteers in all, and they were split equally into two groups: those who smoked and those who did not. During the study period, participants were gathered from the outpatient clinics and the chest department.

Study population

Predetermined inclusion and exclusion criteria were used to choose the participants. According to Global Initiative for Asthma (GINA) requirements, people had to be at least 18 years old and have a verified diagnosis of asthma in order to be eligible [9]. Based on their self-reported and clinically confirmed smoking status, participants were divided into two groups. Participants were excluded if they had undiagnosed or unconfirmed asthma, incomplete clinical records, other respiratory conditions (such as COPD or bronchiectasis), or refused to give informed consent. Through the reduction of potential confounders associated with other pulmonary or systemic disorders, this categorization guaranteed a more accurate comparison between the two groups.

Data collection

A structured clinical interview and a review of medical records were used to collect data. A history of smoking, asthma, and demographic data (age, sex) were recorded. The baseline clinical assessment also included a comprehensive physical examination of each participant.

Clinical and functional assessment

Pulmonary Function Testing

Spirometry was performed using a Smart PFTs Trolley S (Medical Equipment Europe GmbH, Hammelburg, Germany), adhering to American Thoracic Society (ATS)/European Respiratory Society (ERS) guidelines for lung function testing. Each participant underwent pre- and post-bronchodilator spirometry. A short-acting bronchodilator (400 mcg salbutamol via metered-dose inhaler with spacer) was administered, and post-bronchodilator values were recorded 15 minutes later. Each test included a minimum of three acceptable and reproducible maneuvers, with the best effort used for analysis. The following parameters were recorded both before and after bronchodilator administration: VC%, FVC%, FEV₁%, FEV₁/FVC ratio, FEF25-75%, MVV%. These values were compared to standard predicted values to evaluate the degree of airway obstruction and its reversibility.

Ethical considerations

The Ethics Committee of Hassan Ghazzawi Hospital granted ethical permission (DHGH-MBEC-2506). Each method that involved human subjects was carried out in compliance with the Declaration of Helsinki [10]. Prior to data collection, all subjects provided written informed consent. Throughout the study, individuals' anonymity and confidentiality were rigorously protected.

Statistical analysis

Data were analyzed using SPSS version 25 (IBM Corp., Armonk, NY, USA). Continuous variables were expressed as mean ± standard deviation (SD). All pulmonary function parameters, including VC%, FVC%, FEV₁%, FEV₁/FVC ratio, FEF25-75%, and MVV%, were analyzed. To assess the effect of bronchodilator administration within each group (smokers and non-smokers), the paired t-test was used to compare pre- and post-bronchodilator values. To compare parameters between smokers and non-smokers, the independent samples t-test was employed for both pre- and post-bronchodilator values. A p-value of less than 0.05 was considered statistically significant. All tests were two-tailed. Assumptions of normality and equal variances were considered based on the sample size (n = 30 per group), where the Central Limit Theorem supports the use of parametric tests.

## Results

A total of 60 participants were enrolled, consisting of 30 asthmatic smokers and 30 asthmatic non-smokers (Table 1). The mean age of asthmatic smokers was 45.0 ± 10.4 years, which was significantly lower than the mean age of 52.0 ± 8.5 years observed in non-smokers (p = 0.0038). This suggests that the smoker group tended to be younger, which may reflect earlier onset or diagnosis of asthma in smokers. The distribution of sex was comparable between groups, with 23.3% males and 76.7% females among smokers, and 30.0% males and 70.0% females among non-smokers (p = 0.56), indicating that gender differences are unlikely to influence the outcomes in this study.

**Table 1:** Comparison of Age and Sex Between Asthmatic Smokers and Non-Smokers t < 0.05 = statistically significant chi-square < 0.05 = statistically significant

Variable	Smokers (n = 30)	Non-Smokers (n = 30)	value	Test Used
Age (years)	45.0 ± 10.4	52.0 ± 8.5	t value 0.0038 **	Independent t-test
Sex (M)	7 (23.3%)	9 (30.0%)	chi-square value 0.56	Chi-square test
Sex (F)	23 (76.7%)	21 (70.0%)		

In Table 2, the lung volume-related metrics, such as VC, FVC, FEV₁, and expiratory duration results are represented. Before and after using a bronchodilator, these measurements show how much air is expelled during respiratory maneuvers and reveal differences in expiratory effort and ventilatory function between asthmatic smokers and non-smokers.

**Table 2:** Pulmonary volume parameters and expiratory effort measurements between asthmatic smokers and non-smokers before and after bronchodilator BD = bronchodilator, VC = vital capacity, FVC = forced vital capacity, FEV₁ = forced expiratory volume in the first second **Paired t-test used for within-group comparison; Independent t-test used for between-group comparison. t < 0.05 is considered statistically significant.

Group	Pre-BD (Mean ± SD)	Post-BD (Mean ± SD)	Within-Group value	Between-Group value (Pre-BD)	Between-Group value (post-BD)
VC% Smokers (n = 30)	78.3 ± 6.7	80.3 ± 5.8	0.0008 **	< 0.001 **	< 0.001 **
VC% Non-Smokers (n = 30)	88.6 ± 5.6	89.3 ± 5.6	0.072	< 0.001 **	< 0.001 **
FVC% Smokers (n = 30)	68.2 ± 7.3	72.0 ± 7.1	0.0001 **	< 0.001 **	< 0.001 **
FVC% Non-Smokers (n = 30)	78.1 ± 6.9	83.0 ± 6.7	< 0.0001 **	< 0.001 **	< 0.001 **
FEV₁% Smokers (n = 30)	47.2 ± 6.8	60.3 ± 11.5	< 0.0001 **	< 0.0001 **	< 0.0001 **
FEV₁% Non-Smokers (n = 30)	56.3 ± 7.1	73.3 ± 7.4	< 0.0001 **	< 0.001 **	< 0.001 **
Exp Time Smokers (n=30)	3.3 ± 0.2	3.5 ± 0.2	0.00001 **	< 0.001 **	< 0.001 **
Exp Time Non-Smokers (n=30)	3.3 ± 0.4	3.7 ± 0.3	0.00001 **	< 0.001 **	< 0.001 **

The mean VC in asthmatic smokers before bronchodilator use was 78.3 ± 6.7%, which significantly increased to 80.3 ± 5.8% after bronchodilator administration (p = 0.0008), indicating a modest but statistically significant improvement. In contrast, non-smokers had higher baseline VC values of 88.6 ± 5.6%, which increased slightly to 89.3 ± 5.6% post-bronchodilator, though this change was not statistically significant (p = 0.072). When comparing between groups, non-smokers consistently demonstrated significantly higher VC values than smokers at both pre-bronchodilator (p < 0.001) and post-bronchodilator (p < 0.001) time points. These findings suggest that asthmatic smokers have reduced baseline lung volumes and respond more significantly to bronchodilator therapy, whereas non-smokers maintain higher VC levels with minimal post-bronchodilator change (Table 2).

The mean FVC in asthmatic smokers was 68.2 ± 7.3% before bronchodilator administration, which significantly increased to 72.0 ± 7.1% after bronchodilator use (p = 0.0001). In asthmatic non-smokers, the mean FVC improved from 78.1 ± 6.9% to 83.0 ± 6.7%, also showing a statistically significant increase (p < 0.0001). When comparing between groups, non-smokers had significantly higher FVC values than smokers both before and after bronchodilator administration (p < 0.001 for both time points). These findings indicate that while both groups demonstrate bronchodilator responsiveness, non-smokers maintain better baseline ventilatory function and show greater post-bronchodilator improvement compared to smokers (Table 2). Meanwhile, the mean FEV₁ in asthmatic smokers was 47.2 ± 6.8% before bronchodilator use, which significantly improved to 60.3 ± 11.5% after administration (p < 0.0001). Non-smokers had significantly better baseline lung function, with a mean FEV₁ of 56.3 ± 7.1% that increased to 73.3 ± 7.4% post-bronchodilator (p < 0.0001). Between-group comparisons showed that non-smokers had significantly higher FEV₁ values both at baseline and after bronchodilator use (p < 0.0001 for both). These results suggest that while both groups demonstrate bronchodilator responsiveness, non-smokers have better preserved expiratory function. The lower FEV₁ and reduced reversibility in smokers may reflect chronic airway changes, supporting the possibility of airway remodeling or asthma-COPD overlap (Table 2).

The mean expiratory time in the smoker group was 3.3 ± 0.2 seconds before bronchodilator administration and significantly increased to 3.5 ± 0.2 seconds after bronchodilator use (paired t-test, p < 0.0001). In the non-smoker group, expiratory time increased from 3.3 ± 0.4 seconds pre-bronchodilator to 3.7 ± 0.3 seconds post-bronchodilator, also showing a statistically significant improvement (paired t-test, p < 0.0001). Between-group comparisons revealed that while baseline expiratory times were comparable, post-bronchodilator expiratory time was significantly higher in non-smokers than in smokers (independent t-test, p < 0.001 for both pre- and post-bronchodilator). These findings suggest that bronchodilator administration increases expiratory time in both smokers and non-smokers, indicating improved airway patency and reduced airway resistance. However, non-smokers exhibited a greater improvement in expiratory time, particularly after bronchodilator use, which may reflect better baseline lung function and greater reversibility of airflow limitation. Although pre-bronchodilator expiratory time was similar between the groups, the less pronounced post-bronchodilator response in smokers suggests persistent airflow obstruction or decreased lung elasticity, potentially due to chronic smoking-related airway remodeling (Table 2).

The FEV₁/FVC ratio (%), a computed metric based on FVC and FEV₁, is shown in Table 3. It is frequently employed to evaluate airflow restriction and distinguish between restrictive and obstructive lung diseases. The mean FEV₁/FVC ratio in the smoker group before bronchodilator administration was 66.9 ± 5.6, which significantly increased to 76.6 ± 7.4 after bronchodilator use (paired t-test, p < 0.0001). Similarly, in the non-smoker group, the mean FEV₁/FVC ratio increased from 73.3 ± 4.7 pre-bronchodilator to 85.1 ± 4.3 post-bronchodilator (paired t-test, p < 0.0001). Comparison between groups revealed that smokers had a significantly lower FEV₁/FVC ratio than non-smokers both before (independent t-test, p < 0.001) and after bronchodilator administration (independent t-test, p < 0.001). These results demonstrate that smokers have a significantly reduced baseline lung function compared to non-smokers, as indicated by a lower FEV₁/FVC ratio before bronchodilator administration. Both groups exhibited significant improvement in FEV₁/FVC following bronchodilator use, suggesting some degree of reversible airway obstruction. The more pronounced bronchodilator response in smokers indicates that airway obstruction in this group may be at least partially reversible, consistent with conditions such as asthma or COPD with reversible components. Despite improvement, smokers’ post-bronchodilator lung function remained significantly lower than that of non-smokers, highlighting ongoing airway impairment.

**Table 3:** FEV₁/FVC ratio (%) derived pulmonary function parameter between asthmatic smokers and non-smokers BD = bronchodilator, FVC = forced vital capacity, FEV₁ = forced expiratory volume in the first second **Paired t-test used for within-group comparison; Independent t-test used for between-group comparison. t < 0.05 is considered statistically significant.

Group	Pre-BD FEV_1_/FVC (Mean ± SD)	Post-BD FEV_1_/FVC (Mean ± SD)	Within-Group t value	Between-Group t value (Pre-BD)	Between-Group t value (Post-BD)
Smokers (n=30)	66.9 ± 5.6	76.6 ± 7.4	< 0.0001 **	< 0.001 **	< 0.001 **
Non-Smokers (n=30)	73.3 ± 4.7	85.1 ± 4.3	< 0.0001 **	<0.001 **	<0.001 **

Table 4 displays pulmonary function metrics related to flow, such as MVV and FEF25-75. When evaluating small airway function and overall respiratory muscle performance, these markers show the state of VC and airway patency. The mean FEF25-75 in smokers before bronchodilator administration was 34.5 ± 11.3, which significantly increased to 39.1 ± 11.5 after bronchodilator use (paired t-test, p < 0.0001). In non-smokers, the mean FEF25-75 ratio increased from 51.1 ± 8.6 pre-bronchodilator to 57.2 ± 8.7 post-bronchodilator, also showing a statistically significant improvement (paired t-test, p < 0.0001). Between-group comparisons revealed that smokers had significantly lower FEF25-75 values compared to non-smokers both before (independent t-test, p < 0.001) and after bronchodilator administration (independent t-test, p < 0.001).

**Table 4:** Pulmonary flow parameters between asthmatic smokers and non-smokers BD = bronchodilator, MVV = maximal voluntary ventilation, FEF25-75 = forced expiratory flow over the middle one half of the forced vital capacity **Paired t-test used for within-group comparison; Independent t-test used for between-group comparison. t < 0.05 is considered statistically significant.

Group	Pre-BD (Mean ± SD)	Post-BD (Mean ± SD)	Within-Group t-value	Between-Group t-value (Pre-BD)	Between-Group t-value (Post-BD)
FEF25-75 Smokers (n=30)	34.5 ± 11.3	39.1 ± 11.5	0.00001 **	< 0.001 **	< 0.001 **
FEF25-75 Non-Smokers (n=30)	51.1 ± 8.6	57.2 ± 8.7	0.00001 **	< 0.001 **	< 0.001 **
MVV Smokers (n=30)	27.8 ± 12.2	32.7 ± 12.3	0.00001 **	< 0.001 **	< 0.001 **
MVV Non-Smokers (n=30)	42.2 ± 12.2	48.3 ± 12.3	0.00001 **	< 0.001 **	< 0.001 **

These results indicate that smokers have significantly reduced small airway function compared to non-smokers, as shown by the lower baseline FEF25-75 values. Both smokers and non-smokers experienced a significant improvement in FEF25-75 following bronchodilator administration, suggesting partial reversibility of small airway obstruction. However, despite improvement, smokers’ post-bronchodilator FEF25-75 values remained significantly lower than those of non-smokers, reflecting persistent airway impairment in this group, likely due to smoking-related airway damage.

The mean MVV in the smoker group was 27.8 ± 12.2 before bronchodilator administration, which significantly increased to 32.7 ± 12.3 after bronchodilator use (paired t-test, p < 0.0001). In the non-smoker group, MVV increased from 42.2 ± 12.2 pre-bronchodilator to 48.3 ± 12.3 post-bronchodilator, also showing a statistically significant improvement (paired t-test, p < 0.0001). Comparison between the groups demonstrated that smokers had significantly lower MVV values than non-smokers both before (p < 0.001) and after (p < 0.001) bronchodilator administration, as determined by independent t-tests.

These findings indicate that MVV is significantly reduced in smokers compared to non-smokers, both at baseline and after bronchodilator use, suggesting compromised VC due to smoking-related pulmonary changes. While both groups showed statistically significant improvement in MVV post-bronchodilator, the increase was less pronounced in smokers, and their absolute values remained significantly lower than those of non-smokers. This pattern suggests persistent ventilatory limitation in smokers, likely due to airflow obstruction and reduced respiratory muscle efficiency, which may be partially reversible but still indicative of chronic lung damage.

## Discussion

Smokers with asthma had far lower baseline lung function and higher response to bronchodilators, which suggests more reversible airway blockage. In comparison to non-smokers, their VC, FVC, FEV₁, FEV₁/FVC, FEF25-75, and MVV values are lower. Nonsmokers have less bronchodilator responsiveness and better retained lung function. Nonsmokers' post-bronchodilator expiratory times were noticeably longer, indicating longer-lasting airflow, as reported before [11].

Since smoking-related lung damage can occur without obvious symptoms or classic spirometry changes, often missed by traditional diagnostic methods. Our study’s use of detailed pre- and post-bronchodilator spirometry reveals early lung impairments in smokers, which may lead to future respiratory issues. Our study's findings align with several key observations in the literature regarding the detrimental effects of smoking on pulmonary function and the limited reversibility of these impairments in asthmatic smokers.

The bronchodilator response of smokers in our population was notably higher, indicating a larger degree of reversible airway blockage. This could be a result of hyperresponsiveness brought on by smoking that is uncommon in non-smokers. Although there aren't many current studies specifically examining bronchodilator responsiveness stratified by smoking status, Lin and Li's account from 2023 highlights how asthmatic smokers' remodelling process leads to both irreversible and reversible airway alterations [12]. For instance, Smith et al. [13] demonstrated that symptomatic smokers with preserved pulmonary function exhibited increased respiratory exacerbations and airway inflammation, despite normal spirometry results.

Our study's observation that smokers had significantly lower baseline spirometric values compared to non-smokers and exhibited a diminished bronchodilator response corroborates these findings. The reduced improvement in lung function post-bronchodilator in smokers may indicate early, subclinical airway damage that is less responsive to bronchodilators. This is consistent with the concept of "preserved ratio impaired spirometry" (PRISm), where smokers exhibit airflow limitation without meeting the traditional criteria for COPD. According to one study [14], people with significant respiratory symptoms who had retained lung function as shown by spirometry did not see a reduction in respiratory symptoms after receiving simultaneous long-acting bronchodilator therapy. This aligns with our findings that smokers exhibited diminished bronchodilator responsiveness, suggesting early-stage airway damage. Moreover, a comparison of bronchodilator responses using pre-bronchodilator versus predicted normal values is also published. The study emphasised the importance of using predicted normal values for a more accurate assessment of bronchodilator responsiveness, which supports our approach of detailed spirometric assessments [15]. An examination of bronchodilator reversibility in morbidly obese non-smokers found that bronchodilator response varied among individuals, highlighting the need for personalised assessment, which is consistent with our observation of varying bronchodilator responses among asthmatic smokers [16]. A study found that respiratory exacerbations in individuals with a history of smoking but normal spirometry were associated with lung function decline. Experiencing one or more severe exacerbations was associated with increased mortality compared to those with no severe exacerbations [17]. A study examined the effect of a post-bronchodilator FEV₁/FVC ratio of less than 0.7 on COPD diagnosis and treatment. The study found that this criterion may not accurately reflect COPD severity, suggesting the need for alternative diagnostic approaches [18]. In summary, our findings also reinforce well-established observations in the literature: smoking within asthma populations is associated with accelerated decline in lung function, poor asthma control, higher exacerbation rates, and diminished response to inhaled corticosteroids (ICS) [19,20]. Therefore, we would like to highlight the need for regular spirometry screening in smokers, even if asymptomatic, to catch early lung dysfunction. The results suggest that reduced bronchodilator response in smokers suggests treatment should include anti-inflammatory approaches alongside bronchodilators. It also stresses the importance of smoking cessation programs that focus on long-term lung health, not just quitting.

Limitation

There are various restrictions on this study. Due to its cross-sectional design, it is unable to determine causality or monitor changes in lung function over time between smokers with asthma and non-smokers. Additionally, factors that could affect spirometric data, such as the severity of asthma, the length of the illness, or medication adherence, were not taken into consideration in this investigation. Furthermore, a confounding influence might have been introduced because specifics about smoking history, such as the number of packs smoked annually, the length of time smoked, or exposure to secondhand smoke, were not included in the studies. Another drawback is the lack of inflammatory biomarkers (such as eosinophils and fractional exhaled nitric oxide (FeNO)), which would have provided a more thorough understanding of airway inflammation and how smoking affects it. Finally, the study's conclusions can't be applied to larger populations because it was only carried out at one location with a small sample size.

Recommendation

Future studies should use a longitudinal study design to assess smoking's long-term effects on lung function decrease and asthma control to build on these findings. More information about the varying inflammatory responses of asthmatic smokers and non-smokers may be obtained by including inflammatory biomarkers such as FeNO, blood eosinophil counts, and sputum profiles. To inform tailored treatment plans, a thorough smoking history should be part of every asthma patient's routine clinical evaluation. Smokers with asthma may need special management strategies because of their decreased reactivity to bronchodilators and possible remodelling of their airways. To stop more lung damage, it is also crucial to incorporate focused smoking cessation programs into asthma treatment. It is also advised to conduct larger, multi-centre research to validate these findings and improve their generalisability across a range of asthmatic populations.

## Conclusions

This study demonstrates that asthmatic smokers exhibit significantly greater impairment in pulmonary function compared to asthmatic non-smokers, as evidenced by lower baseline spirometric values and a diminished bronchodilator response. These findings suggest that smoking exacerbates airway obstruction and may contribute to fixed airflow limitation, even in individuals already diagnosed with asthma. The reduced bronchodilator reversibility among smokers also indicates a possible overlap with chronic obstructive airway pathology, highlighting the need for careful evaluation and personalised management strategies. Routine spirometric monitoring, including both pre- and post-bronchodilator testing, should be incorporated into the clinical care of asthmatic smokers to detect early functional decline. Additionally, aggressive smoking cessation support and alternative anti-inflammatory interventions may be necessary to optimise outcomes in this high-risk subgroup. Our findings reinforce the harmful interaction between smoking and asthma and emphasise the importance of preventive public health strategies targeting this vulnerable population.
